# Omega-3 and Omega-6 fatty acids and risk of psychotic outcomes in the ALSPAC birth cohort

**DOI:** 10.1016/j.schres.2020.09.018

**Published:** 2020-10

**Authors:** A.D. Thompson, H.J. Jones, J. Heron, J. Hibbeln, S. Sullivan, S. Zammit

**Affiliations:** a Division of Mental Health and Wellbeing, University of Warwick, UK; b Orygen, the Centre of Excellence in Youth Mental Health, 35 Poplar Rd, Parkville, VIC 3250, Australia; c Centre for Academic Mental Health, Bristol Medical School, University of Bristol, Bristol, UK; d NIHR Biomedical Research Centre at University Hospitals Bristol NHS Foundation Trust, University of Bristol, UK; e Laboratory of Membrane Biochemistry and Biophysics, Section of Nutritional Neuroscience, National Institute for Health, Bethesda, USA; f NIHR CLAHRC West, Whitefriars, Lewins Mead, Bristol, UK; g MRC Centre for Neuropsychiatric Genetics and Genomics, Division of Psychological Medicine and Clinical Neurosciences, Cardiff University, Cardiff, UK

**Keywords:** Psychotic experiences, Long chain polyunsaturated fatty acid, Psychosis, Birth cohort, Risk factors

## Abstract

**Background:**

Long chain polyunsaturated fatty acid (PUFA) levels have been implicated in the pathology of psychotic disorders. We investigated the relationship between childhood PUFA levels and later psychotic experiences (PE's) in a large birth cohort.

**Methods:**

Plasma levels of Ω-3 and Ω-6 fatty acids (FA's) were assayed at ages 7 and 16 years. PE's were assessed at ages 12 and 18 years using a semi-structured interview. Primary outcome was any PE's at 18 years; sensitivity analyses examined incident PE's between ages 12 and 18 years, persistent PE's (at 12 and 18) and psychotic disorder at 18 years. Genetic instruments for Ω-3 and Ω-6 were derived and used in a multivariable Mendelian Randomization analysis.

**Results:**

Higher levels of Ω-6 FA's AA, OA and AdA at age 7 years were weakly associated with a reduced risk for PE's at 18 years, however, effect sizes were small and attenuated after adjusting for confounders (strongest evidence for OA; adjusted OR, 0.842; 95% CI, 0.711, 0.998; p, 0.048). Total Ω-6 levels at age 16 years were associated with an increased odds of psychotic disorder at age 18 years. However, there was no association between Ω-6/Ω-3 ratio and psychosis outcomes, nor with genetic instruments of total Ω-3 or Ω-6 levels.

**Conclusions:**

There is no strong evidence that total plasma Ω-3 FA levels or Ω-6/Ω-3 ratios in childhood and mid-adolescence are associated with increased risk for PE's or psychotic disorder, but very marginal evidence that alterations in the Ω-6 pathway at developmental time points might influence risk^2^.

## 1 Introduction

The role of nutritional factors in mental health has recently received greater research focus (Sarris et al., 2015). A recent systematic review reported a consistent trend across studies of a relationship between good-quality diet and better child and adolescent mental health (O'Neil et al., 2014), including common psychiatric disorders such as depression and anxiety, but also psychotic disorders such as schizophrenia. An especially important nutrient group for brain development are the essential fatty acids, including the long chain polyunsaturated fatty acids (PUFAs). Long chain PUFAs such as omega-3 are involved in the neuromodulation of neurotransmitter uptake, degradation and synthesis and receptor binding (Dyall, 2015; Liu et al., 2015). They also enhance cell membrane fluidity and neurogenesis via up regulation of Brain-Derived Neurotrophic Factor (BDNF) (Mischoulon and Freeman, 2013). Omega-3 and omega-6 fatty acids also seem to have distinct roles in the inflammatory pathway (Simopoulos, 2002), with omega-3 PUFAs such as docosahexaenoic acid (DHA) and eicosapentaenoic acid (EPA) involved in the anti-inflammatory pathway, and omega-6 PUFAs such as arachidonic acid (AA) and docosapentaenoic acid (DPA) appearing to be pro-inflammatory (Liu et al., 2015). The balance between omega-6 levels and omega-3 levels has been proposed to be important in a number of inflammatory diseases (Simopoulos, 2002). It can be measured from dietary intake or biological measures and can be calculated by the ratio of mean n-6 and n-3 fatty acid levels (Sheppard and Cheatham, 2013) or of total levels (Thesing et al., 2018). Inflammatory processes have long been proposed as a pathological mechanism in psychosis (Khandaker et al., 2015), supported by evidence from clinical studies of microglial activation (Cannon, 2016), and epidemiological studies of prenatal and childhood infections (Karlsson and Dalman, 2020), autoimmune disorders (Jeppesen and Benros, 2019), and proinflammatory cytokines (Momtazmanesh et al., 2019).

PUFA abnormalities have been found in a number of studies with patients with psychotic disorders. Low levels of omega-3 (and omega-6) fatty acids have also been found in the red cell membranes of patients with schizophrenia (Peet et al., 1995), although this is not consistent across all studies (Medema et al., 2016). Associations between psychotic symptoms and lowered levels of some omega-3 (Docosapentaenoic acid, DPA) and 6 fatty acids (γ-linolenic and docosadienoic acids) in red cell membranes have been found in “at risk for psychosis” groups (Kim et al., 2016). Omega-6/omega-3 dietary intake, possibly reflecting the balance between neuroprotective and proinflammatory pathways, is also altered in both first episode psychosis and those presenting with clinical high risk for psychosis (Pawełczyk et al., 2017). A causal role of PUFA's in development of schizophrenia is supported by a trial that demonstrated a beneficial effect of relatively high dose omega-3 supplementation (750 mg EPA/DHA) on risk of transition to psychosis in a clinical high risk for psychosis sample(Amminger et al., 2010). A more recent replication trial did not demonstrate an significant overall effect (McGorry et al., 2017), however, increases of the n-3 index, EPA, and DHA in this trial predicted less severe psychopathology and better functioning (Amminger et al., 2020). The same group found that PUFA supplementation in a clinical high risk group may act by normalizing intracellular phospholipase A2 activity and δ-6-desaturase-mediated metabolism of omega-3 and omega-6 PUFAs (Smesny et al., 2014). The researchers hypothesized that omega-3 fatty acid supplementation had an effect on cell membrane stability and, through this and other pathways, acted as a neuroprotector (Amminger et al., 2015).

If levels of PUFA's are altered in patients at risk for psychosis and predict transition to psychosis, then it would be useful to investigate levels of long chain PUFA's in childhood to investigate whether levels or ratio differences at an early age are psychosis risk factors. Cohort studies are the strongest observational study design for investigating the effects of PUFA levels on development of psychopathology, including psychotic disorder. As psychotic disorder is a relatively rare outcome, the presence of psychotic experiences, especially if persistent or associated with impairment, may be a good proxy for psychosis risk. Clinically diagnosed psychosis has a lifetime prevalence of around 3% (Perala et al., 2007), but psychotic experiences and sub-clinical expressions are estimated to occur in approximately 7.5–17% of the general population (Kelleher et al., 2012a; Zammit et al., 2013). This broader spectrum of psychosis can also result in significant distress, functional impairment, increased suicidal behaviour (Kelleher et al., 2012b) and increases the risk of developing a full-blown psychotic disorder in adulthood (Fisher et al., 2013; van Os et al., 1999) especially in individuals whose psychotic experiences persist over time (Dominguez et al., 2011; Kaymaz et al., 2012).

One study in a large cohort of Swedish women aged 30–49 found that the intake of fatty acids at baseline (as measured by a food frequency questionnaire) was related to the levels of self-reported psychotic experiences a decade later (Hedelin et al., 2010). This was the case for both omega-3 and omega-6 dietary fatty acid levels, but the relationship was non-linear and a high fatty acid intake level was also associated with an increased risk of psychotic experiences as well as low levels. This study was in an older population, with potential for residual confounding and used a dietary questionnaire, not biological levels, thus being prone to measurement error (Hedelin et al., 2010). There have been no reports investigating childhood PUFA levels and psychotic experiences.

To address some of these issues we aimed to investigate the association between childhood blood levels of omega-3 and omega-6 fatty acids levels and late adolescent risk of developing psychotic experiences and other psychosis outcomes including psychotic disorder in a large birth cohort. This included investigating genetic proxies of these levels using Mendelian Randomization to address residual confounding and reverse causation. We specifically hypothesized that an altered omega-6/omega-3 ratio in childhood would be associated with an increase in late adolescent psychotic experiences.

## 2 Methods

### 2.1 Participants

The Avon Longitudinal Study of Parents and Children (ALSPAC) is a UK birth cohort study (Boyd et al., 2013; Fraser et al., 2013). Pregnant women resident in Avon District with an expected delivery date between 1st April 1991 and 31st December 1992 were approached to participate in the study, leading to the inclusion of 14,775 live births with 14,701 of these alive at 1 year of age. This analysis is based on 4720 children who completed the Psychosis-LIKe Symptoms interview (PLIKSi) at approximately 18 years of age. Ethical approval for the study was obtained from the ALSPAC Ethics and Law Committee and the Local Research Ethics Committees (http://www.bristol.ac.uk/alspac/researchers/research-ethics/). Consent for biological samples has been collected in accordance with the Human Tissue Act (2004). Informed consent for the use of data collected via questionnaires and clinics was obtained from participants following the recommendations of the ALSPAC Ethics and Law Committee at the time.

### 2.2 Outcome variables

#### 2.2.1 Primary outcome

##### 2.2.1.1 Psychotic experiences

The PLIKSi (Horwood et al., 2008; Zammit et al., 2013) is a semi-structured face-to-face interview, rated according to guidelines for the Schedules for Clinical Assessment in Neuropsychiatry version 2.0 (SCAN 2.0), which elicits key psychotic experiences covering hallucinations (visual and auditory), delusions (spied on, persecution, thoughts read, reference, control, grandiosity and other unspecified delusions), and experiences of thought interference (thought broadcasting, insertion and withdrawal). The PLIKS interview was carried out in the cohort at the age of 12 and the age of 18. The average kappa values for inter-rater and test-retest reliability were 0.83 and 0.76 at the age of 18 (Zammit et al., 2013). Further detail on the instrument is available elsewhere (Zammit et al., 2013) as well as further detail on this data (Horwood et al., 2008; Zammit et al., 2013). The primary outcome was ≥1 of the PLIKSi experiences *suspected* or *definitely* present at age 18 and *not* attributable to sleep, fever or substance use.

#### 2.2.2 Sensitivity analyses

We also examined three other psychosis outcomes, including some that are potentially more clinically meaningful but less common. These were: a) at least 1 suspected or definite psychotic experience incident by age 18 (i.e. not present at 12), b) at least 1 suspected or definite psychotic experience present at both age 12 and age 18 (persistent PLIKS), and c) psychotic disorder at age 18. We classified individuals as having a psychotic disorder if they reported definite psychotic experiences not attributable to the effects of sleep or fever that had occurred at least once per month over the previous 6 months and either caused severe distress, had a markedly negative impact on social or occupational function, or led to help seeking (see Zammit et al., 2013).

### 2.3 Independent variables

#### 2.3.1 Omega-3/6 PUFA levels

Overall plasma PUFA levels and levels of individual omega-3 and omega-6 fatty acids were available from non-fasting blood samples taken from the children at mean 7.5 years (5576). From these samples, plasma was obtained after centrifugal separation and frozen immediately. All samples were stored at −70 °C, thawed once to obtain a 100 μl aliquot, shipped airfreight on dry ice to Rockville MD and thawed a second time for analysis. Transmethylation of lipids with acetyl chloride and methanol was performed using a simplified method based on the Lepage and Roy procedure (Lepage and Roy, 1986) using a high throughput automated method (Masood and Salem, 2007). Internal calibration was conducted by adding internal standards to each assay. A second standard was used to quantify the exact amount of internal standard in every batch for ongoing assay of experimental variability. Freedom Evo Instrument 200 (TECAN Trading AG, Switzerland) was utilised for the automatic transmethylation and extraction of fatty acids employing the customised control and automation software (EVOware V.2.0, SP1, Patch3). Gas chromatography 6890 Plus LAN system (Agilent Technologies, Inc., Santa Clara, California, USA) coupled with a fused-silica, narrow-bored DB-FFAP capillary column (Agilent 127-32H2, 15 m × 0.1 mm ID×0.1 μm film thickness) which was used for chromatographic separation of the fatty acid methyl esters as reported previously (Masood and Salem, 2007). The assay was linear in the range of 1–600 μg/ml plasma. The within and between day imprecision was 3.26 ± 1.2% and 2.95 ± 1.6% for fatty acid concentrations. Assays were undertaken in 2009–2010. In all, 23 FAs were measured including 12 polyunsaturates (Golding et al., 2012).

Summary fatty acids levels and some specific individual fatty acids (DHA and LA) were also measured at age 16 years using a high-throughput Nuclear Magnetic Resonance (NMR) metabolomics platform (Soininen et al., 2015; Soininen et al., 2009). Metabolite data were assessed on fasting (minimum 6-h) plasma. All fatty acid levels were expressed as percentage of total fatty acids and standardized before analyses.

**Fig. 1:**
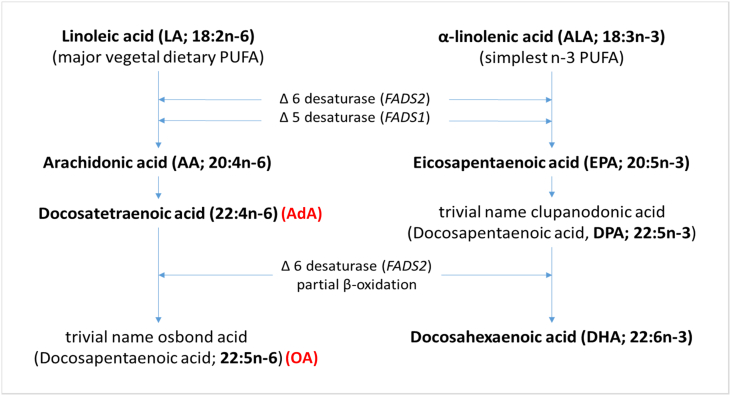
Omega 3 and Omega 6 metabolic pathway (from Calder, 2017).

A summary of the available fatty acid measures are shown in Table 1 and the omega-3 and omega-6 metabolic pathways are shown in Fig. 1.

**Table 1:** Omega 3 (n-3) and omega 6 (n-6) fatty acids measures available in ALSPAC at ages 7 and 16 years.

Measure (as % of total fatty acids)		Age	
		7 years	16 years
n-6	LA	✓	✓
	AA	✓	
	AdA	✓	
	OA	✓	
	Total n3		✓
n-3	ALA	✓	
	EPA	✓	
	DPA	✓	
	DHA	✓	✓
	Total n6		✓
ratio	AA:EPA	✓	
	n6:n3	✓	✓

Note: LA, linoleic acid (18:2n-6); AA, arachidonic acid (20:4n-6); AdA, docosatetraenoic acid (trivial name adrenic acid; 22:4n-6); OA, omega-6 docosapentaenoic acid (trivial name osbond acid; 22:5n-6); total n6, total levels of omega-6 fatty acids; ALA, α-linolenic acid (18:3n-3); EPA, eicosapentaenoic acid (20:5n-3); DPA omega-3 docosapentaenoic acid (22:5n-3); DHA docosahexaenoic acid (22:6n-3); total n3, total levels of omega-3 fatty acids; n6:n3, ratio of total levels of omega-6 to total levels of omega-3 fatty acids.

### 2.4 Genetic data

Following quality control assessment and imputation, genetic data was available for 8252 ALSPAC individuals. For further information on the genetic data please refer to the online Supplementary methods.

#### 2.4.1 Fatty acid genetic instruments

Due to competition for the same elongases and desaturases during omega-6 and omega-3 PUFA biosynthesis, it is difficult to disentangle the specific effects of omega-3 from omega-6 on an outcome. One approach that can be used to attempt to overcome this is by using genetic instruments for omega-3 and omega-6 FA levels within a multivariable analysis. This method, known as MultiVariable Mendelian Randomization (MVMR), is an extension of single exposure Mendelian randomization whereby genetic variants that reliably predict one exposure variable, but do not influence the outcome through any other pathway, are used as instrumental variables in estimating the true causal effect of the exposure on the outcome (Davey Smith and Hemani, 2014).

Genetic instruments were derived using summary statistics from the recent Kettunen et al. (2016) meta-analysis of genome-wide association studies (GWAS) of blood levels of DHA, LA, total omega-3 and total omega-6 fatty acids measured using a high-throughput NMR metabolomics platform in up to 24,925 individuals (Kettunen et al., 2016). Based on the GWAS meta-analysis summary statistics, a list of SNPs that were associated with total omega-3 and total omega-6 fatty acid levels at a genome-wide level of significance (*p* value ≤5e^−8^) was extracted. Further information on the genetic instruments please refer to the online Supplementary methods and Supplementary Table 1.

### 2.5 Confounders

For the non-genetic analyses, the following confounders were able to be considered due to their potential influence on the exposure and outcome: *Gender*; *Ethnic group*; *Highest Parental Social Class*; *Maternal Education* (A levels/degree or equivalent (higher education) v O-level/CSE's (Certificate of Secondary Education - lowest UK school-leaving qualifications)); *Birth Weight*; *Family Adversity Index* (FAI) multiple family risk factors were assessed during pregnancy with the Family Adversity Index (Bowen et al., 2005); *Body Mass Index* (*BMI*) *at 7 and at 16 years* (The child's BMI was calculated at the same clinic when the blood sample were taken for the FA analysis); *Energy intake at 7 and at 13 years* (The child's overall energy intake was calculated from the completion of the Food Frequency Questionnaire as part of the interview ages 7 and 13 years). Please see Supplementary methods for further details of confounders and Supplementary Table 2 for number of individuals with available data on each confounder and the proportion who also had available psychotic experience data.

### 2.6 Statistical analyses

#### 2.6.1 Observational analyses

Logistic regression was performed to assess the association between fatty acid plasma levels in ALSPAC at age 7 and age 16 years and psychotic experiences outcomes at age 18 years. Results are presented for unadjusted and adjusted analyses, where potential confounders have been included as covariates within the logistic regression. We additionally tested the associations between each genetic instrumental variable for omega-3 and omega-6 FA fatty acid levels in ASLPAC and psychotic experiences at age 18 years using logistic regression. All statistical analyses were performed using Stata (Version 15.1; StataCorp LP, College Station, TX, USA).

#### 2.6.2 Mendelian randomization

A two-stage least squares MVMR was implemented using the ivreg2 (Baum et al., 2002) command in STATA v14.2 with robust standard errors. The strength of the instrumental variables used in the analysis was assessed using the Sanderson-Windmeijer conditional F test of excluded instruments (Sanderson and Windmeijer, 2016) which tests if the genetic instruments strongly and jointly predict both exposures in the outcome model. Genetic instruments with an F-statistic of 10 or less are generally deemed to be weak.

## 3 Results

### 3.1 Description of the sample

The frequencies of psychotic experiences in the cohort and in those with PUFA data are shown in Table 2. Rates were similar in those with and without PUFA data. The summary statistics for the fatty acid levels at 7 and 16 years are shown in Supplementary Table 3. PUFA levels were available for 5565 children at age 7 years and 3356–3360 children at age 16 years.

**Table 2:** Prevalence of psychotic experiences at age 18 years.

Psychotic experience measure	N	N (%) with PE	N with fatty acid data at age 7 years	N (%) with PE	N with fatty acid data at age 16 years	N (%) with PE
Definite/suspected versus none	4718	432 (9.16)	2844	229 (8.05)	2465	208 (8.44)
Incident psychotic experiences versus none^a^	3513	205 (5.84)	2308	140 (6.07)	1981	110 (5.55)
Persistent psychotic experiences versus none^b^	4058	134 (3.30)	2631	61 (2.32)	2299	77 (3.35)
Psychotic disorder versus none^c^	4718	79 (1.67)	2844	43 (1.51)	2465	38 (1.54)

a Denotes individuals with definite or suspected psychotic experiences at age 18 years and no definite or suspected psychotic experiences at age 12 years.

b Denotes individuals with definite or suspected psychotic experiences at age 12 years and age 18 years.

c Psychotic disorder defined by psychotic experiences not attributable to the effects of sleep or fever that had occurred at least once per month over the previous 6 months and either caused severe distress, had a markedly negative impact on social or occupational function, or led to help seeking.

### 3.2 Association of PUFA plasma levels at 7 years and psychotic experiences at 18 years

In the unadjusted analyses there was weak evidence that higher levels of omega-6 AA, AdA and OA were associated with a decreased odds of psychotic experiences at age 18 years (AA: odds ratio (OR), 0.853; 95% confidence interval (CI), 0.728, 0.999; p, 0.049; AdA: OR, 0.826; 95% CI, 0.702, 0.972; p, 0.021; OA: OR, 0.822; 95% CI, 0.697, 0.970; p, 0.020). The CI included the null for all but one of the adjusted associations between fatty acid levels at age 7 years (OA) and psychotic experiences at age 18 years (OA: adjusted OR, 0.842; 95% CI, 0.711, 0.998; p, 0.048) (see Table 3).

**Table 3:** Unadjusted and adjusted association between fatty acid measures at age 7 years and psychotic experiences at age 18 years.

Outcome	Exposure	Unadjusted		Adjusted^a^		N	N (%) with PE
		OR (95% CIs)	P	OR (95% CIs)	P		
Definite/suspected psychotic experiences versus none	LA	0.993 (0.851, 1.159)	0.931	1.001 (0.856, 1.172)	0.986	2310	174 (7.53)
	AA	0.853 (0.728, 0.999)	0.049	0.873 (0.742, 1.026)	0.100		
	AdA	0.826 (0.702, 0.972)	0.021	0.855 (0.723, 1.012)	0.068		
	OA	0.822 (0.697, 0.970)	0.020	0.842 (0.711, 0.998)	0.048		
	Total n6	0.938 (0.805, 1.094)	0.415	0.954 (0.817, 1.114)	0.553		
	ALA	1.043 (0.896, 1.214)	0.586	1.038 (0.891, 1.210)	0.633		
	EPA	0.997 (0.853, 1.164)	0.967	0.987 (0.843, 1.156)	0.871		
	DPA	0.982 (0.841, 1.146)	0.813	0.995 (0.848, 1.167)	0.948		
	DHA	0.978 (0.841, 1.139)	0.776	1.016 (0.871, 1.186)	0.835		
	Total n3	0.996 (0.855, 1.160)	0.956	1.020 (0.874, 1.191)	0.803		
	AA:EPA	0.890 (0.758, 1.044)	0.152	0.912 (0.777, 1.072)	0.265		
	n6:n3	0.968 (0.828, 1.132)	0.688	0.956 (0.813, 1.124)	0.585		

Note: OR, odds ratio per standard deviation increase in fatty acid level; 95% CI, 95% confidence interval; P, p-value for association between fatty acid levels at age 7 years and psychotic experiences at age 18 years; LA, linoleic acid (18:2n-6); AA, arachidonic acid (20:4n-6); AdA, docosatetraenoic acid (trivial name adrenic acid; 22:4n-6); OA, omega-6 docosapentaenoic acid (trivial name osbond acid; 22:5n-6); total n6, total levels of omega-6 fatty acids; ALA, α-linolenic acid (18:3n-3); EPA, eicosapentaenoic acid (20:5n-3); DPA omega-3 docosapentaenoic acid (22:5n-3); DHA docosahexaenoic acid (22:6n-3); total n3, total levels of omega-3 fatty acids; n6:n3, ratio of total levels of omega-6 to total levels of omega-3 fatty acids.

a Adjusted for gender; ethnic group; highest parental social class; maternal education; birth weight; family adversity index, body mass index at age 7 years; energy intake at age 7 years.

### 3.3 Association of fatty acids blood levels at 16 years and psychotic experiences at 18 years

In the unadjusted analyses higher DHA levels were associated with a decreased risk of psychotic experiences (OR, 0.871; 95% CI 0.736, 1.032; p, 0.110) but the CI's include the null and this was further attenuated when adjusted for potential confounders (see Table 4).

**Table 4:** Unadjusted and adjusted association between fatty acid measures at age 16 years and psychotic experiences at age 18 years.

Outcome	Exposure	Unadjusted		Adjusted^a^		N	N (%) with PE
		OR (95% CIs)	P	OR (95% CIs)	P		
Definite/suspected psychotic experiences versus none	LA	1.001 (0.850, 1.180)	0.990	0.991 (0.840, 1.170)	0.917	1974	159 (8.05)
	Total n6	0.944 (0.802, 1.111)	0.488	0.938 (0.797, 1.106)	0.447		
	DHA	0.871 (0.736, 1.032)	0.110	0.882 (0.740, 1.051)	0.162		
	Total n3	0.918 (0.779, 1.083)	0.312	0.946 (0.799, 1.120)	0.518		
	n6:n3	1.080 (0.926, 1.259)	0.330	1.050 (0.895, 1.231)	0.550		

Note: OR, odds ratio per standard deviation increase in fatty acid level; 95% CI, 95% confidence interval; P, p-value for association between fatty acid levels at age 16 years and psychotic experiences at age 18 years; LA, linoleic acid (18:2n-6); AA, arachidonic acid (20:4n-6); AdA, docosatetraenoic acid (trivial name adrenic acid; 22:4n-6); OA, omega-6 docosapentaenoic acid (trivial name osbond acid; 22:5n-6); total n6, total levels of omega-6 fatty acids; ALA, α-linolenic acid (18:3n-3); EPA, eicosapentaenoic acid (20:5n-3); DPA omega-3 docosapentaenoic acid (22:5n-3); DHA docosahexaenoic acid (22:6n-3); total n3, total levels of omega-3 fatty acids; n6:n3, ratio of total levels of omega-6 to total levels of omega-3 fatty acids.

a Adjusted for gender; ethnic group; highest parental social class; maternal education; birth weight; family adversity index, body mass index at age 16 years; energy intake at age 13 years.

### 3.4 Sensitivity analyses

We performed sensitivity analyses examining different psychosis outcomes including more stringent/clinically-relevant psychosis outcomes. When the outcome excluded those who reported psychotic experiences at age 12 years, (incident psychotic experiences) an association was seen for higher levels of omega-6 AA, AdA and OA and there was weak evidence that lower AA:EPA ratio at age 7 years increased the odds of reporting psychotic experiences (Supplementary Table 4). Higher LA and total omega-6 levels at age 16 years were associated with an increased odds of psychotic disorder at age 18 years, with evidence slightly weakening in the adjusted analysis, especially for LA (Supplementary Table 5).

### 3.5 Association of fatty acids genetic instruments and psychotic experiences

The allelic scores (weighted and unweighted) acted as stronger instruments for total omega-3 and total omega-6 fatty acid measures at age 16 years than using the individuals SNPs as instruments (Table 5); however, no Sanderson-Windmeijer F-statistic exceeded 10 indicating that the genetic instruments were weak. There was no strong evidence that instrumental variables for levels of omega-3 or omega-6 were associated with psychotic experiences at 18 years of age, nor the other secondary psychosis outcomes (Table 5 and Supplementary Table 6).

**Table 5:** Multivariable Mendelian randomization results showing associations between total omega-3 and omega-6 fatty acids at age 16 years and definite/suspected psychotic experiences at age 18 years.

Exposure	N	OR (LCI, UCI)	P	SW F	SW F P
*Instruments* = *unweighted allele scores*					
Total omega-3	1922	1.14 (0.83, 1.57)	0.421	8.41	0.004
Total omega-6		0.91 (0.75, 1.10)	0.326	2.39	0.122

*Instruments* = *weighted allele scores*					
Total omega-3	1922	1.17 (0.82, 1.68)	0.384	6.94	0.009
Total omega-6		0.91 (0.77, 1.09)	0.307	2.73	0.099

*Instruments* = *individual SNPs*					
Total omega-3	543	1.04 (0.84, 1.30)	0.698	0.96	0.511
Total omega-6		1.00 (0.95, 1.05)	0.904	1.26	0.206

Note: OR, odds ratio; LCI, lower 95% confidence level; UCI, upper 95% confidence level; SW, Sanderson-Windmeijer conditional F test of excluded instruments.

## 4 Discussion

The study aimed to investigate the association between childhood plasma levels of long chain PUFAs and development of psychotic experiences in late adolescence. Overall, omega-3 and omega-6 fatty acids levels taken at 7 and 16 years were not strongly associated with any of the psychotic outcomes examined. When examining our primary outcome, there was weak evidence that higher levels of the omega-6 fatty acid OA and AdA at age 7 years were associated with a decreased risk of psychotic experiences but only the OA association survived adjustment of confounders and effect sizes were small. When examining other psychosis outcomes, there was stronger evidence of association between OA, AdA and also AA at age 7 years, and of a higher AA (omega-6):EPA (omega-3) ratio at this age, with reduced risk of incident psychotic experiences between ages 12 and 18 years. There was also very weak evidence that total omega-6 fatty acid levels at 16 years were associated with increased risk of psychotic disorder. In the Mendelian randomization analysis there was no evidence of association between GWAS-based instrumental variables for total omega-3 and omega-6 levels and psychotic experiences at 18 years.

The majority of studies in patients with established psychosis have found a reduction in PUFA levels, especially DHA, DPA and AA (Hoen et al., 2013). Authors have suggested that these results could be secondary to antipsychotic treatment although some studies have found reduced levels in antipsychotic naive patients (Reddy et al., 2004). However, it is unclear if these PUFA changes in these populations with an established psychotic disorder reflect a propensity to poorer response to inflammation or even an early response to inflammation or a marker of oxidative stress. Cohort studies or studies in at risk groups are therefore key to try to disentangle these effects but there is very limited data on PUFA levels in patients prior to the onset of psychosis or psychotic experiences. The only cohort study in the extant literature of which we are aware reported that higher levels of omega-3 and omega-6 as measured by dietary intake were associated with a reduced risk of later psychotic experiences (Hedelin et al., 2010). The associations were reverse J-shaped with the strongest reduced risk for an intermediate intake of fish or PUFA (Hedelin et al., 2010).

In our study, there was no evidence that higher plasma levels of the omega-3 PUFA DHA in late adolescence may be protective against broad psychotic experiences. However, whilst higher levels of a number omega-6 PUFAs in childhood appeared to be protective against psychotic experiences, we also found in our sensitivity analyses that higher levels of omega-6 PUFAs in adolescence appeared to increase the risk for developing a psychotic disorder. These findings are not consistent with the suggestion that overactivity of the pro-inflammatory omega-6 pathway may be propsychotic and overactivity of the anti-inflammatory omega-3 pathway may be antipsychotic (Amminger et al., 2010), although the structure of the omega-6/omega-3 pathways suggests that relationships of these PUFAs with psychopathology may be highly complex and that studying their ratio may be more informative. The omega-6/omega-3 plasma ratio (expressed through dietary levels) that has been reported to be altered/elevated in patients at risk for psychosis (Pawełczyk et al., 2017) compared to healthy controls, was not associated with the psychosis outcomes in our study.

There are a number of possible explanations for this. Firstly, PUFA levels may be differentially important at specific periods of development. For example, any potential neuroprotective balance of omega-6/omega-3 PUFAs might be particularly important in guarding against psychosis risk in the adolescent brain when synaptic pruning is occurring in brain areas believed to be key in psychosis, such as the prefrontal cortex and limbic structures. This could also be why omega-3 fatty acid supplementation may be relatively more effective in younger at-risk groups than in populations with established psychosis, who are generally older.

Secondly, it could be that any early dysfunction in PUFA metabolism is actually located in different places in the pathway and later deficiencies represent a potential compensatory or downstream effect. The omega-6 and omega-3 pathways are not independent of each other and any enzyme deficiency or dietary deficiency will have an effect on other parts of the pathway. This is the reason why we chose not to look at individual PUFA levels in our MR approach but examined the overall effects of the pathways using total levels.

Thirdly, the plasma levels at ages 7 and 16 years were measured differently, with levels at age 16 years taken after 6 h fasting and levels at age 7 years non-fasting. We are also aware that we have only examined PUFA levels in isolation and these differences might represent the downstream effect of other overlapping processes such as eicosanoids and other inflammatory pathways. For example, a previous study in this cohort reported higher levels of inflammatory cytokines Interleukin 6 and C Reactive Protein to be related to psychosis (Khandaker et al., 2014), and there are a number of other factors that influence these pathways (Horrobin et al., 1994). We also did not examine omega 9 PUFA levels, as these were not available in the cohort. More research is needed into the interplay between PUFA levels, PUFA metabolism, pro and anti-inflammatory eicosanoids and other inflammatory proteins and risk for developing psychotic symptoms and psychotic disorder.

The results of the MR approach also did not demonstrate any clear associations between these genetic proxies of PUFA levels and psychotic experiences. This leads us to have more certainty that there is not a clear relationship that is confounded by a factor we were not able to adjust for. MR is a useful tool in areas where there are clear genetic variants in biological pathways (Davey Smith and Hemani, 2014). We have examined overview levels of total PUFA levels in isolation in this MR analysis and not specific omega 3 FAs such as EPA or DHA that are used in supplementation, nor omega-6/omega-3 ratios.

### 4.1 Strengths and limitations

The strengths of the research are the large dataset, the robust measurements both of psychotic experiences and PUFA levels (at 2 time points), and the ability to adjust for multiple confounders. There is likely to be no or very minimal antipsychotic use prior to the measure of PLIKS at 18 years (this is a non-clinical population). We have also performed an MR analysis to attempt to specifically address unspecified confounding and reverse causation effects.

Although the sample size is large, there is a considerable amount of missing data from the original cohort and there are relatively small numbers for some of the secondary psychosis outcomes, meaning the results for these outcomes should be interpreted with this caveat. For secondary outcomes such as psychotic disorder that sample is still below the expected age of peak incidence. There a number of other potential confounders such as cannabis use, cigarette smoking and childhood trauma as well as information on dietary supplementation and other general nutritional factors that were not adjusted for. The numbers are also relatively small for the MR analysis.

The use of plasma fatty acid levels and not red blood cell membrane (RBC) levels or brain cell membrane levels is also a potential weakness. The membrane hypothesis of schizophrenia posits that there are potential alterations in the cell membrane structure (Horrobin, 1999; Horrobin et al., 1994) and we are unable to examine this in this cohort. Although there is often considerable correlation between plasma PUFA levels and those measured in RBC membranes (Sun et al., 2007), plasma levels are more sensitive to fluctuations in diet and recent intake whereas RBC levels reflect long term fatty acid intake (Harris and Thomas, 2010). There are some measures of RBC PUFA's in the ALSPAC cohort although the sample with data is small (just over 500) and the samples were of cord blood and may be mixed fetal and maternal samples; therefore, a meaningful analysis was not possible. It is also necessary to mention that the assays used to measure PUFA levels differ by age (gas chromatography at 7 years vs. NMR at 16 years). Although units were standardized before analyses, the sensitivity of gas chromatography is superior to NMR. The PUFA levels at 7 years were also non-fasting as opposed to the levels at 16 years being 6 h fasting. We note the MR analysis is likely to give a longer-term proxy of FA levels as an intermediate variable that is not susceptible to fluctuation in diet and intake. We have also not measured eicosanoid levels in this analysis and how these relate to the PUFA levels.

### 4.2 Clinical implications

There is no clear evidence of specific alterations in PUFA metabolism prior to the onset of psychotic symptoms or psychotic disorder in our findings. If PUFA's are protective against psychosis inset, any potential mechanism of protection and the timing of any protection remains unclear or whether changes seen in psychosis risk samples are a consequence of pathological processes in a psychotic state; the results of current and ongoing trials in at risk for psychosis subjects (Kane and Correll, 2017) may shed further light on this.

### 4.3 Conclusions

In this cohort study did not find evidence of childhood PUFA levels being strongly associated with psychotic experiences and other psychosis outcomes in late adolescence. This may reflect that alterations may be a state phenomena or a timing issue with levels being especially important during times when brain inflammatory and anti-inflammatory processes are key.

## Declaration of competing interest

The authors report no conflicts of interest.

## Appendix A Supplementary data

Supplementary material Image 1
