# Supplementary material for: Omega-3 and Omega-6 fatty acids and risk of psychotic outcomes in the ALSPAC birth cohort
Source: Schizophr Res. 2020 Oct;224:108–15. doi: 10.1016/j.schres.2020.09.018 (PMC7738752; doi:10.1016/j.schres.2020.09.018)
Supplement: Supplementary file 1 — Supplementary material [file mmc1.docx]

Supplementary Table 1**.** Independent genetic variants used as genetic instruments for total omega-3 and total omega-6 fatty acid levels

| **SNP** | **Chr:BP** | **A1/A2** | **Freq A1**  **in ALPSAC** | **omega-3 beta^a^** | **omega-3 p value^a^** | **omega-6 beta^b^** | **omega-6 p value^b^** | **Used in allelic score** |
| --- | --- | --- | --- | --- | --- | --- | --- | --- |
| rs638714 | 1:62906489 | T/G | 0.36 | -0.052 | 2.53E-04 | -0.077 | 4.78E-08 | omega-6 |
| rs6547692 | 2:27734972 | A/G | 0.55 | -0.078 | 5.54E-10 | -0.058 | 3.62E-06 | omega-3 |
| rs144064722 | 4:73406173 | G/A | 0.02 | 0.154 | 1.04E-04 | 0.237 | 2.29E-09 | omega-6 |
| rs2032885 | 5:74613918 | G/C | 0.54 | -0.036 | 5.55E-03 | -0.072 | 3.27E-08 | omega-6 |
| rs149804 | 11:61538606 | G/A | 0.39 | 0.085 | 6.91E-11 | 0.008 | 5.51E-01 | omega-3 |
| rs174475 | 11:61672645 | C/G | 0.46 | 0.087 | 6.59E-12 | 0.017 | 1.67E-01 | omega-3 |
| rs10466588 | 11:116610249 | G/A | 0.08 | 0.096 | 3.27E-06 | 0.138 | 1.76E-11 | omega-6 |
| rs180327 | 11:116623659 | T/C | 0.65 | -0.056 | 9.40E-06 | -0.092 | 2.26E-13 | omega-6 |
| rs567496 | 15:58665952 | A/T | 0.39 | -0.040 | 2.22E-03 | -0.080 | 1.20E-09 | omega-6 |
| rs493258 | 15:58687880 | C/T | 0.53 | -0.045 | 2.75E-04 | -0.078 | 3.91E-10 | omega-6 |
| rs12914626 | 15:58738423 | T/C | 0.71 | -0.050 | 2.25E-04 | -0.086 | 2.50E-10 | omega-6 |
| rs17231506 | 16:56994528 | T/C | 0.32 | 0.005 | 7.10E-01 | 0.076 | 3.20E-08 | omega-6 |
| rs11668477 | 19:11195030 | G/A | 0.21 | -0.028 | 9.28E-02 | -0.095 | 6.57E-09 | omega-6 |
| rs8100204 | 19:19393714 | A/G | 0.11 | -0.122 | 7.05E-10 | -0.104 | 1.43E-07 | omega-3 |
| rs189741280 | 19:19624481 | G/A | 0.01 | -0.204 | 7.38E-09 | -0.138 | 9.31E-05 | omega-3 |
| rs150617279 | 19:20139234 | A/T | 0.06 | -0.121 | 7.09E-09 | -0.058 | 5.80E-03 | omega-3 |
| rs6857 | 19:45392254 | T/C | 0.15 | 0.039 | 1.64E-02 | 0.115 | 1.50E-12 | omega-6 |
| rs72654473 | 19:45414399 | A/C | 0.10 | -0.036 | 1.50E-01 | -0.156 | 5.97E-10 | omega-6 |
| rs157594 | 19:45425175 | G/T | 0.57 | 0.040 | 4.81E-03 | 0.096 | 1.91E-11 | omega-6 |

**Note:** SNP, single nucleotide polymorphism; Chr, chromosome; BP, base position; A1/A2, effect allele/alternative allele; Freq A1, frequency of the effect allele in the Avon Longitudinal Study of Parents and Children (ALSPAC) birth cohort.

^a^ SNP association effect size and p-value for omega-3 fatty acid levels as discovered in the Kettunen et al. (2016) fatty acid genome-wide association study meta-analysis ^1^

^b^ SNP association effect size and p-value for omega-6 fatty acid levels as discovered in the Kettunen et al. (2016) fatty acid genome-wide association study meta-analysis ^1^

Supplementary Table 2. Total number of individuals with data available for confounder measures and proportion with psychotic experience data available at age 18 years.

| **Confounder** | **Category / Age** | **Total N** | **Category %** | **N with PE measure  (% with PE)**† | **Missing PE measure**†† **%** |
| --- | --- | --- | --- | --- | --- |
| **Categorical measures** | | | | | |
| Gender | female | 7219 | 48.60 | 2665 (10.43) | 63.08 |
|  | male | 7635 | 51.40 | 2052 (7.50) | 73.12 |
|  | total | 14854 | 100.00 | 4717 (9.16) | 68.24 |
| Ethnic group | Bangladeshi | 7 | 0.06 | 1 (0.00) | 85.71 |
|  | Black African | 11 | 0.09 | 2 (0.00) | 81.82 |
|  | Black Caribbean | 76 | 0.61 | 21 (4.76) | 72.37 |
|  | Black non-Africa, non-Caribbean | 44 | 0.35 | 8 (0.00) | 81.82 |
|  | Chinese | 30 | 0.24 | 9 (0.00) | 70.00 |
|  | Indian | 54 | 0.44 | 16 (12.5) | 70.37 |
|  | Pakistani | 22 | 0.18 | 4 (0.00) | 81.82 |
|  | White | 12075 | 97.37 | 4186 (9.03) | 65.33 |
|  | Other | 82 | 0.66 | 29 (20.69) | 64.63 |
|  | total | 12401 | 100.00 | 4276 (9.05) | 65.52 |
| Highest parental social class | professional / managerial | 6328 | 55.09 | 2635 (7.36) | 58.36 |
|  | non-professional /  non-managerial | 5158 | 44.91 | 1481 (11.41) | 71.29 |
|  | total | 11486 | 100.00 | 4116 (8.82) | 64.17 |
| Maternal education – highest qualification | CSE | 2527 | 20.23 | 497 (14.08) | 80.33 |
|  | vocational | 1229 | 9.84 | 311 (9.97) | 74.69 |
|  | O level | 4327 | 34.64 | 1463 (10.66) | 66.19 |
|  | A level | 2801 | 22.42 | 1193 (5.95) | 57.41 |
|  | degree | 1609 | 12.88 | 827 (7.62) | 48.60 |
|  | total | 12493 | 100.00 | 4291 (9.11) | 65.65 |
| **Continuous measures** | | | | | |
| Birth weight | - | 13895 | - | 4384 (9.19) | 68.45 |
| Family adversity index | - | 12536 | - | 4326 (8.95) | 65.49 |
| Body mass index | 7 years | 8201 | - | 4033 (8.33) | 50.82 |
|  | 16 years | 5415 | - | 3816 (8.41) | 29.53 |
| Energy intake | 7 years | 7279 | - | 3720 (8.04) | 48.89 |
|  | 13 years | 6101 | - | 3928 (8.43) | 35.62 |

**Note:** PE, psychotic experiences; CSE, Certificate of Secondary Education.

† Denotes individuals with data available on the confounder measure and psychotic experiences at age 18 years, with percentage of individuals with definite or suspected psychotic experiences denoted in the parentheses.

†† Denotes the percentage of individuals with data available on the confounder measure but with missing data on psychotic experiences at age 18 years.

Supplementary Table 3. Summary statistics of fatty acid levels (non-transformed, expressed as percentages of total fatty acid content) at ages 7 and 16 years

|  | **Fatty Acid** | **N** | **Mean** | **Std. Dev.** | **Min** | **Max** |
| --- | --- | --- | --- | --- | --- | --- |
| **Age 7 years** | | | | | | |
| n-6 | LA | 5565 | 30.597 | 3.175 | 16.631 | 44.417 |
|  | AA | 5565 | 6.436 | 1.322 | 2.538 | 12.275 |
|  | AdA | 5565 | 0.248 | 0.051 | 0.091 | 0.526 |
|  | OA | 5565 | 0.186 | 0.049 | 0.016 | 0.479 |
|  | Total n6 | 5565 | 37.467 | 3.815 | 20.486 | 51.562 |
| n-3 | ALA | 5565 | 0.719 | 0.280 | 0.216 | 2.898 |
|  | EPA | 5565 | 0.644 | 0.211 | 0.118 | 4.687 |
|  | DPA | 5565 | 0.627 | 0.139 | 0.223 | 1.475 |
|  | DHA | 5565 | 1.889 | 0.524 | 0.599 | 5.079 |
|  | Total n3 | 5565 | 3.879 | 0.796 | 1.497 | 10.839 |
| ratio | AA:EPA | 5565 | 10.713 | 3.330 | 1.424 | 42.283 |
|  | n-6:n-3 | 5565 | 10.010 | 2.087 | 3.037 | 24.119 |
| **Age 16 years** | | | | | | |
| n-6 | LA | 3356 | 27.753 | 2.708 | 15.080 | 38.650 |
|  | Total n6 | 3359 | 33.989 | 2.545 | 20.400 | 42.220 |
| n-3 | DHA | 3360 | 1.039 | 0.267 | 0.254 | 3.095 |
|  | Total n3 | 3360 | 3.183 | 0.609 | 1.414 | 8.438 |
| ratio | n-6:n-3 | 3357 | 11.044 | 2.184 | 3.232 | 26.330 |

**Note:** LA, linoleic acid (18:2n-6); AA, arachidonic acid (20:4n-6); AdA, docosatetraenoic acid (trivial name adrenic acid; 22:4n-6); OA, omega-6 docosapentaenoic acid (trivial name osbond acid; 22:5n-6); total n6, total levels of omega-6 fatty acids; ALA, α-linolenic acid (18:3n-3); EPA, eicosapentaenoic acid (20:5n-3); DPA omega-3 docosapentaenoic acid (22:5n-3); DHA docosahexaenoic acid (22:6n-3); total n3, total levels of omega-3 fatty acids; n6:n3, ratio of total levels of omega-6 to total levels of omega-3 fatty acids.

Supplementary Table 4. Unadjusted and adjusted association between fatty acid measures at age 7 years and other psychosis outcomes at age 18 years

|  | | **Unadjusted** | | **Adjusted**† | |  |  |
| --- | --- | --- | --- | --- | --- | --- | --- |
| **Outcome** | **Exposure** | **OR (95% CIs)** | **P** | **OR (95% CIs)** | **P** | **N** | **N (%) with PE** |
| Incident psychotic experiences versus none^a^ | LA | 0.970 (0.798, 1.177) | 0.755 | 0.972 (0.799, 1.183) | 0.780 | 1904 | 106 (5.57) |
|  | AA | 0.770 (0.628, 0.944) | 0.012 | 0.776 (0.630, 0.956) | 0.017 |  |  |
|  | AdA | 0.774 (0.625, 0.957) | 0.018 | 0.784 (0.630, 0.975) | 0.029 |  |  |
|  | OA | 0.770 (0.620, 0.956) | 0.018 | 0.782 (0.627, 0.976) | 0.029 |  |  |
|  | Total n6 | 0.890 (0.735, 1.079) | 0.235 | 0.897 (0.739, 1.089) | 0.274 |  |  |
|  | ALA | 0.993 (0.811, 1.215) | 0.943 | 0.997 (0.813, 1.222) | 0.975 |  |  |
|  | EPA | 1.051 (0.870, 1.270) | 0.604 | 1.043 (0.859, 1.265) | 0.672 |  |  |
|  | DPA | 0.942 (0.772, 1.148) | 0.553 | 0.937 (0.764, 1.149) | 0.531 |  |  |
|  | DHA | 0.964 (0.794, 1.171) | 0.713 | 0.996 (0.819, 1.211) | 0.967 |  |  |
|  | Total n3 | 0.976 (0.802, 1.189) | 0.811 | 0.996 (0.817, 1.216) | 0.972 |  |  |
|  | AA:EPA | 0.786 (0.630, 0.979) | 0.032 | 0.801 (0.642, 0.999) | 0.049 |  |  |
|  | n6:n3 | 0.967 (0.794, 1.179) | 0.742 | 0.954 (0.779, 1.168) | 0.649 |  |  |
| Persistent psychotic experiences versus none^b^ | LA | 0.995 (0.750, 1.319) | 0.971 | 1.017 (0.762, 1.357) | 0.910 | 2165 | 49 (2.26) |
|  | AA | 0.926 (0.696, 1.233) | 0.601 | 0.978 (0.730, 1.309) | 0.880 |  |  |
|  | AdA | 0.951 (0.711, 1.271) | 0.733 | 1.036 (0.767, 1.399) | 0.819 |  |  |
|  | OA | 0.949 (0.709, 1.270) | 0.724 | 1.019 (0.752, 1.381) | 0.905 |  |  |
|  | Total n6 | 0.969 (0.733, 1.282) | 0.827 | 1.007 (0.758, 1.336) | 0.963 |  |  |
|  | ALA | 1.052 (0.800, 1.383) | 0.719 | 1.043 (0.791, 1.376) | 0.765 |  |  |
|  | EPA | 0.938 (0.694, 1.268) | 0.677 | 0.922 (0.678, 1.254) | 0.605 |  |  |
|  | DPA | 1.011 (0.763, 1.341) | 0.938 | 1.038 (0.775, 1.391) | 0.801 |  |  |
|  | DHA | 0.915 (0.685, 1.221) | 0.545 | 0.976 (0.731, 1.305) | 0.872 |  |  |
|  | Total n3 | 0.946 (0.709, 1.262) | 0.706 | 0.986 (0.737, 1.318) | 0.922 |  |  |
|  | AA:EPA | 0.918 (0.686, 1.227) | 0.563 | 0.963 (0.725, 1.279) | 0.792 |  |  |
|  | n6:n3 | 0.998 (0.750, 1.327) | 0.987 | 0.982 (0.729, 1.322) | 0.903 |  |  |

*Supplementary Table 4. continued*

| Psychotic disorder versus none^c^ | LA | 1.000 (0.701, 1.425) | 0.998 | 1.039 (0.721, 1.497) | 0.838 | 2310 | 31 (1.34) |
| --- | --- | --- | --- | --- | --- | --- | --- |
|  | AA | 1.117 (0.789, 1.582) | 0.532 | 1.187 (0.830, 1.697) | 0.347 |  |  |
|  | AdA | 0.947 (0.660, 1.359) | 0.768 | 1.051 (0.726, 1.520) | 0.793 |  |  |
|  | OA | 0.877 (0.604, 1.272) | 0.489 | 0.933 (0.635, 1.371) | 0.724 |  |  |
|  | Total n6 | 1.037 (0.727, 1.478) | 0.841 | 1.094 (0.761, 1.573) | 0.626 |  |  |
|  | ALA | 0.922 (0.627, 1.356) | 0.680 | 0.891 (0.602, 1.318) | 0.564 |  |  |
|  | EPA | 1.146 (0.849, 1.549) | 0.373 | 1.149 (0.852, 1.551) | 0.362 |  |  |
|  | DPA | 1.273 (0.914, 1.772) | 0.153 | 1.385 (0.980, 1.957) | 0.065 |  |  |
|  | DHA | 1.039 (0.741, 1.456) | 0.825 | 1.080 (0.769, 1.518) | 0.657 |  |  |
|  | Total n3 | 1.087 (0.777, 1.520) | 0.628 | 1.116 (0.798, 1.560) | 0.523 |  |  |
|  | AA:EPA | 0.737 (0.487, 1.115) | 0.148 | 0.753 (0.493, 1.151) | 0.191 |  |  |
|  | n6:n3 | 0.833 (0.568, 1.222) | 0.350 | 0.825 (0.554, 1.228) | 0.343 |  |  |

**Note:** OR, odds ratio per standard deviation increase in fatty acid level; 95% CI, 95% confidence interval; P, p-value for association between fatty acid levels at age 7 years and psychotic experiences at age 18 years; LA, linoleic acid (18:2n-6); AA, arachidonic acid (20:4n-6); AdA, docosatetraenoic acid (trivial name adrenic acid; 22:4n-6); OA, omega-6 docosapentaenoic acid (trivial name osbond acid; 22:5n-6); total n6, total levels of omega-6 fatty acids; ALA, α-linolenic acid (18:3n-3); EPA, eicosapentaenoic acid (20:5n-3); DPA omega-3 docosapentaenoic acid (22:5n-3); DHA docosahexaenoic acid (22:6n-3); total n3, total levels of omega-3 fatty acids; n6:n3, ratio of total levels of omega-6 to total levels of omega-3 fatty acids.

^a^ Denotes individuals with definite or suspected psychotic experiences at age 18 years and no definite or suspected psychotic experiences at age 12 years

^b^ Denotes individuals with definite or suspected psychotic experiences at age 12 years and age 18 years

^c^ Psychotic disorder defined by psychotic experiences not attributable to the effects of sleep or fever that had occurred at least once per month over the previous 6 months and either caused severe distress, had a markedly negative impact on social or occupational function, or led to help seeking

† Adjusted for gender; ethnic group; highest parental social class; maternal education; birth weight; family adversity index, body mass index at age 7 years; energy intake at age 7 years, with the exception of analyses of incident psychotic experiences and persistent psychotic experiences where ethnic group wasn’t used as a covariate as it did not vary with the outcome.

Supplementary Table 5. Unadjusted and adjusted association between fatty acid measures at age 16 years and psychotic experiences at age 18 years

|  | | **Unadjusted** | | **Adjusted**† | |  |  |
| --- | --- | --- | --- | --- | --- | --- | --- |
| **Outcome** | **Exposure** | **OR (95% CIs)** | **P** | **OR (95% CIs)** | **P** | **N** | **N (%) with PE** |
| Incident psychotic experiences versus none^a^ | LA | 0.968 (0.783, 1.196) | 0.762 | 0.970 (0.783, 1.201) | 0.778 | 1663 | 90 (5.41) |
|  | Total n6 | 0.874 (0.710, 1.075) | 0.203 | 0.877 (0.711, 1.081) | 0.218 |  |  |
|  | DHA | 0.858 (0.688, 1.070) | 0.174 | 0.862 (0.685, 1.084) | 0.205 |  |  |
|  | Total n3 | 0.829 (0.663, 1.037) | 0.101 | 0.846 (0.674, 1.064) | 0.153 |  |  |
|  | n6:n3 | 1.145 (0.943, 1.390) | 0.173 | 1.127 (0.922, 1.379) | 0.244 |  |  |
| Persistent psychotic versus none^b^ | LA | 1.007 (0.774, 1.311) | 0.959 | 0.996 (0.765, 1.297) | 0.975 | 1919 | 58 (3.02) |
|  | Total n6 | 0.995 (0.764, 1.295) | 0.968 | 0.988 (0.759, 1.287) | 0.930 |  |  |
|  | DHA | 0.934 (0.718, 1.215) | 0.613 | 0.965 (0.736, 1.265) | 0.795 |  |  |
|  | Total n3 | 1.000 (0.774, 1.291) | 0.999 | 1.045 (0.806, 1.355) | 0.740 |  |  |
|  | n6:n3 | 1.014 (0.788, 1.306) | 0.911 | 0.972 (0.749, 1.262) | 0.833 |  |  |
| Psychotic disorder versus none^c^ | LA | 1.502 (1.034, 2.183) | 0.033 | 1.442 (0.987, 2.107) | 0.059 | 1974 | 28 (1.42) |
|  | Total n6 | 1.676 (1.114, 2.522) | 0.013 | 1.619 (1.068, 2.454) | 0.023 |  |  |
|  | DHA | 0.816 (0.546, 1.222) | 0.324 | 0.852 (0.554, 1.312) | 0.468 |  |  |
|  | Total n3 | 0.939 (0.644, 1.370) | 0.745 | 1.029 (0.695, 1.524) | 0.886 |  |  |
|  | n6:n3 | 1.243 (0.896, 1.724) | 0.192 | 1.161 (0.817, 1.648) | 0.405 |  |  |

**Note:** OR, odds ratio per standard deviation increase in fatty acid level; 95% CI, 95% confidence interval; P, p-value for association between fatty acid levels at age 16 years and psychotic experiences at age 18 years; LA, linoleic acid (18:2n-6); AA, arachidonic acid (20:4n-6); AdA, docosatetraenoic acid (trivial name adrenic acid; 22:4n-6); OA, omega-6 docosapentaenoic acid (trivial name osbond acid; 22:5n-6); total n6, total levels of omega-6 fatty acids; ALA, α-linolenic acid (18:3n-3); EPA, eicosapentaenoic acid (20:5n-3); DPA omega-3 docosapentaenoic acid (22:5n-3); DHA docosahexaenoic acid (22:6n-3); total n3, total levels of omega-3 fatty acids; n6:n3, ratio of total levels of omega-6 to total levels of omega-3 fatty acids.

^a^ Denotes individuals with definite or suspected psychotic experiences at age 18 years and no definite or suspected psychotic experiences at age 12 years

^b^ Denotes individuals with definite or suspected psychotic experiences at age 12 years and age 18 years

^c^ Psychotic disorder defined by psychotic experiences not attributable to the effects of sleep or fever that had occurred at least once per month over the previous 6 months and either caused severe distress, had a markedly negative impact on social or occupational function, or led to help seeking

† Adjusted for gender; ethnic group; highest parental social class; maternal education; birth weight; family adversity index, body mass index at age 16 years; energy intake at age 13 years.

Supplementary Table 6**:** Multivariable Mendelian randomization results showing associations between total omega-3 and omega-6 fatty acids at age 16 years and psychotic experiences at age 18 years

| **Exposure** | **Outcome** | **N** | **OR (LCI, UCI)** | **P** | **SW F** | **SW F P** |
| --- | --- | --- | --- | --- | --- | --- |
| ***Instruments = unweighted allele scores*** | | | | | | |
| Total omega-3 | Incident psychotic experiences versus none^a^ | 1629 | 0.90 (0.72, 1.12) | 0.343 | 8.97 | 0.003 |
| Total omega-6 |  |  | 0.98 (0.87, 1.11) | 0.767 | 2.77 | 0.096 |
| Total omega-3 | Persistent psychotic versus none^b^ | 1889 | 1.16 (0.94, 1.43) | 0.179 | 10.32 | 0.001 |
| Total omega-6 |  |  | 0.95 (0.85, 1.06) | 0.367 | 2.66 | 0.103 |
| Total omega-3 | Psychotic disorder versus none^c^ | 1992 | 1.02 (0.93, 1.12) | 0.727 | 8.41 | 0.004 |
| Total omega-6 |  |  | 1.00 (0.94, 1.06) | 0.945 | 2.39 | 0.122 |
| ***Instruments = weighted allele scores*** | | | | | | |
| Total omega-3 | Incident psychotic experiences versus none^a^ | 1629 | 0.89 (0.69, 1.14) | 0.353 | 6.78 | 0.009 |
| Total omega-6 |  |  | 0.98 (0.89, 1.09) | 0.765 | 3.23 | 0.073 |
| Total omega-3 | Persistent psychotic versus none^b^ | 1889 | 1.19 (0.93, 1.53) | 0.159 | 8.23 | 0.004 |
| Total omega-6 |  |  | 0.96 (0.87, 1.05) | 0.362 | 3.16 | 0.076 |
| Total omega-3 | Psychotic disorder versus none^c^ | 1992 | 1.03 (0.93, 1.15) | 0.554 | 6.94 | 0.009 |
| Total omega-6 |  |  | 1.00 (0.95, 1.05) | 0.996 | 2.73 | 0.099 |
| ***Instruments = individual SNPs*** | | | | | | |
| Total omega-3 | Incident psychotic experiences versus none^a^ | 441 | 1.08 (0.86, 1.36) | 0.498 | 0.74 | 0.765 |
| Total omega-6 |  |  | 0.98 (0.94, 1.02) | 0.347 | 1.34 | 0.159 |
| Total omega-3 | Persistent psychotic versus none^b^ | 508 | 0.93 (0.80, 1.10) | 0.415 | 1.11 | 0.338 |
| Total omega-6 |  |  | 1.02 (0.99, 1.05) | 0.267 | 1.39 | 0.132 |
| Total omega-3 | Psychotic disorder versus none^c^ | 543 | 0.96 (0.88, 1.05) | 0.366 | 0.96 | 0.511 |
| Total omega-6 |  |  | 1.01 (0.98, 1.03) | 0.709 | 1.26 | 0.206 |

**Note:** OR, odds ratio; LCI, lower 95% confidence level; UCI, upper 95% confidence level; SW, Sanderson-Windmeijer conditional F test of excluded instruments

^a^ Denotes individuals with definite or suspected psychotic experiences at age 18 years and no definite or suspected psychotic experiences at age 12 years

^b^ Denotes individuals with definite or suspected psychotic experiences at age 12 years and age 18 years

^c^ Psychotic disorder defined by psychotic experiences not attributable to the effects of sleep or fever that had occurred at least once per month over the previous 6 months and either caused severe distress, had a markedly negative impact on social or occupational function, or led to help seeking

Supplementary Figure 1. Association between fatty acid measures and definite/suspected psychotic experiences at age 18 years

**Note:** SD, standard deviation; LA, linoleic acid (18:2n-6); AA, arachidonic acid (20:4n-6); AdA, docosatetraenoic acid (trivial name adrenic acid; 22:4n-6); OA, omega-6 docosapentaenoic acid (trivial name osbond acid; 22:5n-6); total n6, total levels of omega-6 fatty acids; ALA, α-linolenic acid (18:3n-3); EPA, eicosapentaenoic acid (20:5n-3); DPA omega-3 docosapentaenoic acid (22:5n-3); DHA docosahexaenoic acid (22:6n-3); total n3, total levels of omega-3 fatty acids; n6:n3, ratio of total levels of omega-6 to total levels of omega-3 fatty acids; error bars denote upper and lower 95% confidence interval.

**Supplementary methods**

**Genetic data**

Genetic data were acquired using the Illumina HumanHap550 quad genome-wide single nucleotide polymorphism (SNP) genotyping platform from 9912 participants. Individuals were excluded from further analysis on the basis of gender mismatches, minimal or excessive heterozygosity, disproportionate levels of individual missingness (>3%), evidence of cryptic relatedness (>5% of alleles identical by descent), and being of non-European ancestry (assessed by multidimensional scaling analysis including HapMap 2 individuals). SNPs with a minor allele frequency (MAF) of < 1%, Impute2 information quality metric of < 0.8, a call rate of < 95% or evidence for violations of Hardy-Weinberg equilibrium (*p* value < 5 x 10^-7^) were removed. Imputation of the target data was performed using Impute V2.2.2 against the 1000 genomes reference panel (Phase 1, Version 3; all polymorphic SNPs excluding singletons), using 2186 reference haplotypes (including non-Europeans). Following quality control assessment and imputation, genetic data was available for 8252 ALSPAC individuals.

**Fatty acid genetic instruments**

Genetic instruments were derived using summary statistics from the recent Kettunen et al. (2016) meta-analysis of genome-wide association studies (GWAS) of blood levels of DHA, LA, total omega-3 and total omega-6 fatty acids measured using a high-throughput NMR metabolomics platform in up to 24,925 individuals (Kettunen et al. 2016). Based on the GWAS meta-analysis summary statistics, a list of SNPs that were associated with total omega-3 and total omega-6 fatty acid levels at a genome-wide level of significance (p value ≤ 5e^-8^) was extracted. This list was then harmonised to the ALSPAC genetic data (based on SNP availability and equivalence of alleles) and pruned for linkage disequilibrium based on a r^2^ ≥ 0.1, 3000 kb window size and 1000 genomes reference panel (Phase 1, Version 3) using the PLINK (v1.9) (Purcell et al., 2007; Chang et al. 2015) ‘indep-pairwise’ command. This resulted in a final list of 19 independent SNPs that were associated with total omega-3 and/or total omega-6 fatty acid levels at a genome-wide level of significance (Supplementary Table 1). For the MVMR analysis, SNPs were either used as individual instruments, where each ALSPAC individual had 0, 1 or 2 fatty acid level increasing alleles, or summarised into 2 allelic scores (one for total omega-3 and one for total omega-6) based on the sum of the number of PUFA level increasing alleles each ALSPAC individual possessed. Unweighted and weighted (by discovery GWAS effect sizes) allelic scores were generated using the PLINK ‘score’ command.

**Confounders**

For the non-genetic analyses, the following confounders were able to be considered due to their potential influence on both the exposure and outcome:

*Sociodemographic factors:*

*Gender*; *Ethnic group*; *Highest Parental Social Class; Maternal Education*

A number of socio-demographic variables were included as potential confounders. Questionnaires completed by the parents during the antenatal period provided information on: child’s ethnic background (non-white vs. white); parental social class based on the higher of the mother or partner’s occupational social class (using the 1991 Office of Population Census and Statistics (OPCS) classification (Office of Population Census and Statistics (Office of Population Censuses and Surveys, 1991) and dichotomised into manual and non-manual); Maternal Education was also dichotomised (A levels/degree or equivalent (higher education) v O-level/CSE’s (Certificate of Secondary Education - lowest UK school-leaving qualifications))

*Other confounding factors*

*Birth Weight; Body Mass Index (BMI) at 7 and at 16 (*The child’s BMI was calculated at the same clinic when the blood sample were taken for the FA analysis); *Energy intake at 7 and at 13 (*The child’s overall energy intake was calculated from the completion of the Food Frequency Questionnaire as part of the interview aged 7 and 13). Finally, a *Family Adversity Index* ((FAI) indicating multiple family risk factors were assessed during pregnancy with the Family Adversity Index (Bowen et al., 2005)*.* The FAI consists of 17 items taken from questionnaires that were administered throughout pregnancy (8 weeks gestation, 12 weeks gestation, 18 weeks gestation and 32 weeks gestation). The index was based on a series of measures describing aspects of family functioning covering: young maternal age (<21 years) at first pregnancy / child birth; Housing (e.g. inadequacy: crowding index / periods of homelessness); Financial difficulties; problematic partner relationship, maternal affective disorder (depression, anxiety, suicidality), substance abuse of drugs or involvement in crime (i.e. in trouble with police/convictions). If adversity was present in an item this was rated as 1 and then totaled across the 17 items.

**References**

**1.** Kettunen J, Demirkan A, Wurtz P, et al. Genome-wide study for circulating metabolites identifies 62 loci and reveals novel systemic effects of lpa. *Nat Commun.* 2016;7.

**2.** Purcell S, Neale B, Todd-Brown K, et al. Plink: A tool set for whole-genome association and population-based linkage analyses. *Am J Hum Genet.* 2007;81:559-575.

**3.** Chang CC, Chow CC, Tellier LCAM, Vattikuti S, Purcell SM, Lee JJ. Second-generation plink: Rising to the challenge of larger and richer datasets. *Gigascience.* 2015;4.

4. Office of Population Censuses and Surveys, *Standard Occupational Classification*. 1991, London: Her Majesty's Stationery Office.

5. Bowen, E., Heron, J, Waylen, A, Wolke, D., *Domestic violence risk during and after pregnancy: findings from a British longitudinal study.* Bjog: An International Journal of Obstetrics and Gynaecology., 2005. 112(8):1083-1089.
